# Sclérose tubéreuse de Bourneville: importance de l’anamnèse et de la clinique

**DOI:** 10.11604/pamj.2018.29.147.14941

**Published:** 2018-03-13

**Authors:** Jawad El-Azhari, Naoufal Hjira

**Affiliations:** 1 Service de Dermatologie-vénérologie, Hôpital Militaire d’Instruction Mohammed V, Rabat, Maroc

**Keywords:** Sclérose tubéreuse de Bourneville, acné, épilepsie, Tuberous sclerosis complex, acne, epilepsy

## Abstract

Tuberous sclerosis complex (TSC) is a neurocutaneous multisystemic syndrome most commonly affecting the skin, the brain, the kidneys, the lungs and the eyes. The diagnosis is based on clinical characteristics; 90% of patients have skin lesions and seizures are the initial symptom in 80% of patients. Patients with STB often show a high incidence of neuropsychiatric symptoms, including mental retardation, autism and learning difficulties. Epilepsy and brain and renal tumors are treated with aggressive therapeutic and surgical approaches. We report the case of a 13-year old girl, presenting with a 1-year history of acne resistant to therapy. Clinical examination showed symmetric grouped red papules with smooth surface on the central face and the chin (A). The objective clinical examination also showed frontal fibrous plaques (A), achromic macules on the upper limbs and grayish tumors in the periungual folds of the toes (B). During a new interview, patient’s mother reported that these lesions had their onset when the patient was 5 years old, manifesting as seizures at the age of 2 years and disappearing at the age of 6 years, with good psychomotor development. The girl had a history of consanguinity but she had no other family history. Based on these major criteria, the diagnosis of Tuberous sclerosis complex was made. The patient underwent MRI of the brain showing intensely enhanced multiple bilateral subependymal and intraventricular nodules as well as signal abnormalities in the subcortical white matter at the level of the frontal, parietal and temporal area and linear abnormalities in the subcortical white matter. Thoracoabdominopelvic CT scan showed liver and renal lesions suggesting angiomyolipomas. Ophthalmologic examination, electrocardiogram and cardiac ultrasound were normal. Vascular laser treatment of angiofibromas, annual clinical monitoring and paraclinical monitoring every 3 years were proposed to the patient.

## Image en médecine

La sclérose tubéreuse de Bourneville (STB) est un syndrome neurocutané dont l'atteinte multisystémique affecte le plus souvent la peau, le cerveau, les reins, les poumons et les yeux. Le diagnostic est basé sur les caractéristiques cliniques; les lésions cutanées sont présentes chez 90% des patients et les crises convulsives sont la présentation initiale chez 80% des patients. Les patients atteints de STB présentent souvent une incidence élevée de symptômes neuropsychiatriques, y compris un retard mental, l'autisme et des difficultés d'apprentissage. L'épilepsie et les tumeurs cérébrales et rénales sont gérées de manière agressive par des traitements médicaux et chirurgicaux. Nous rapportons le cas d'une fille de 13 ans, qui vient consulter pour une acné résistante à un traitement mené depuis une année. A l'examen clinique, présence de papules rouges à surface lisse de disposition symétrique centrofaciale et mentonnière (A). L'examen objective aussi des plaques fibreuses frontales (A), des macules achromiques des membres supérieurs, des tumeurs grisâtres des sillons péri-unguéaux des orteils (B). En réinterrogeant la maman, l'apparition de ces lésions remonte à l'âge de 5 ans, avec notion de crises convulsives à l'âge de 2 ans disparues à l'âge de 6 ans, avec un bon développent psychomoteur, et une notion de consanguinité des parents, sans autres antécédents familiaux, ce qui présentait suffisamment de critères majeurs pour retenir le diagnostique de Sclérose Tubéreuse de Bournonville. Devant ce tableau clinique l'examen était complété par une IRM cérébrale ayant objectivé de multiples nodules sous-épendymaires et intra-ventriculaires bilatéraux prenant fortement le contraste ainsi que des anomalies de signal de la substance blanche sous corticale au niveau frontal, pariétal et temporal, et des anomalies de signal linéaires de la substance blanche sous corticale, la TDM thoraco-abdomino-pelvienne a montré des lésions hépatiques et rénales en faveur d'angiomyolipomes. L'examen ophtalmologique, l'électrocardiogramme et l'échographie cardiaque étaient normaux. On a proposé un traitement par Laser vasculaire pour les angiofibromes, une surveillance clinique annuelle et paraclinique tous les 3 ans.

**Figure 1:**
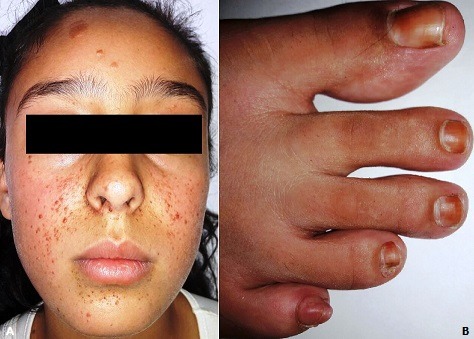
A) angiofibromes centro-faciaux et mentonniers, et plaques fibreuses frontales; B) fibromes périunguéaux

